# Negative affect, affect regulation, and food choice: A value-based decision-making analysis

**DOI:** 10.1192/j.eurpsy.2021.325

**Published:** 2021-08-13

**Authors:** D. O’Leary, A. Brytek-Matera, J. Gross

**Affiliations:** 1 Department Of Psychology, University of Chicago Booth School of Business, Chicago, United States of America; 2 Katowice Faculty Of Psychology, SWPS University of Social Sciences and Humanities, Katowice, Poland; 3 Department Of Psychology, Stanford University, Stanford, United States of America

**Keywords:** dietary food choice task, affect regulation, eating behavior, negative affect

## Abstract

**Introduction:**

Research has shown that negative affect leads to unhealthy eating, the top cause of death in the United States.

**Objectives:**

This project examined whether AR (Affect Regulation) can be applied to incidental negative affect to improve eating behavior.

**Methods:**

We conducted four studies.

**Results:**

In Studies 1 and 2 (n=80), we developed a autobiographical negative affect induction, showed that it induces negative affect, and demonstrated that participants can learn to downregulate this negative affect. In Study 3 (n=40), participants completed a three-phase dietary food choice task. In phase 1, participants made food choices under neutral conditions. In phase 2, participants made food choices after receiving the negative affect induction from Studies 1 and 2. In phase 3, participants made food choices while downregulating the negative affect caused by the induction. In phase 2, participants placed less importance on health (b=-0.15, z=-5.99, p<.001) when making food choices than under neutral conditions (phase 1). In phase 3, participants successfully downregulated their negative affect (b=-1.2, t=-22.01, p<.001) and placed the same level of importance on health when making food choices as in phase 1, indicating that AR applied to incidental affect is an effective method for improving eating behavior. In Study 4 (n=120), we pre-registered and replicated our findings from Study 3. In addition, we fit drift-diffusion models to participants reaction time data and show that these results extent to the by-participant weights participants place on health when making food choices.

**Conclusions:**

These results are a step towards scalable AR interventions to improve eating behavior.

**Disclosure:**

No significant relationships.

